# Complete genome sequence of *Pseudomonas corrugata* strain RM1-1-4, a stress protecting agent from the rhizosphere of an oilseed rape bait plant

**DOI:** 10.1186/s40793-017-0278-7

**Published:** 2017-11-09

**Authors:** Christin Zachow, Henry Müller, Christina M. Laireiter, Ralf Tilcher, Gabriele Berg

**Affiliations:** 1 Austrian Centre of Industrial Biotechnology (ACIB GmbH), Petersgasse 14, 8010 Graz, Austria; 2 Institute of Environmental Biotechnology, Graz University of Technology, Petersgasse 12, 8010 Graz, Austria; 3 KWS SAAT SE, Grimsehlstraße 31, 37555 Einbeck, Germany

**Keywords:** Pseudomonas corrugata, *Sphagnum magellanicum*-treated seeds, Rhizosphere of bait plant, Stress protection, Detoxification systems, Biocontrol, Plant growth promotion

## Abstract

10.1601/nm.2592 strain RM1-1-4 is a rhizosphere colonizer of oilseed rape. A previous study has shown that this motile, Gram-negative, non-sporulating bacterium is an effective stress protecting and biocontrol agent, which protects their hosts against abiotic and biotic stresses. Here, we announce and describe the complete genome sequence of *P. corrugata* RM1-1-4 consisting of a single 6.1 Mb circular chromosome that encodes 5189 protein coding genes and 85 RNA-only encoding genes. Genome analysis revealed genes predicting functions such as detoxifying mechanisms, stress inhibitors, exoproteases, lipoproteins or volatile components as well as rhizobactin siderophores and spermidine. Further analysis of its genome will help to identify traits promising for stress protection, biocontrol and plant growth promotion properties.

## Introduction

10.1601/nm.2592 Roberts and Scarlett (1981) emend. Sutra belongs to the genus 10.1601/nm.2552 sensu stricto and it is one of the few non fluorescent 10.1601/nm.2552 species. 10.1601/nm.2592 strain RM1-1-4 was isolated from the oilseed rape rhizosphere grown in the greenhouse, whose seeds were treated with the microbial community associated with the moss *Sphagnum magellanicum* [1]. *Sphagnum* mosses form bog ecosystems under low-nutrient and extreme conditions supported by their microbiota [2]. RM1-1-4 was selected as stress protecting agent coping high salt concentrations, reactive oxygen species and desiccation [1]. As it has a broad antagonistic spectrum exhibiting antifungal activity against phytopathogenic fungi (Ascomycota: *Alternaria alternata*, *Botrytis cinerea*, *Sclerotinia sclerotiorum*, *Verticillium dahliae* and Basidiomycota: *Rhizoctonia solani* AG2-2IIIB, *Sclerotium rolfsii*), it is a promising candidate for biocontrol purposes. The activity putatively base on the production of exoenzymes and the emission of antimicrobial volatile organic compounds.

In this report, we summarize the complete genome sequence and annotation of RM1-1-4. We also describe its genomic properties revealing multifaceted plant beneficial features. The genome sequence of RM1-1-4 and its comparison with related published genomes will provide a framework for further functional studies of its abiotic and biotic stress protecting effectiveness in plant and rhizosphere competence.

## Organism information

### Classification and features

**Fig. 1:**
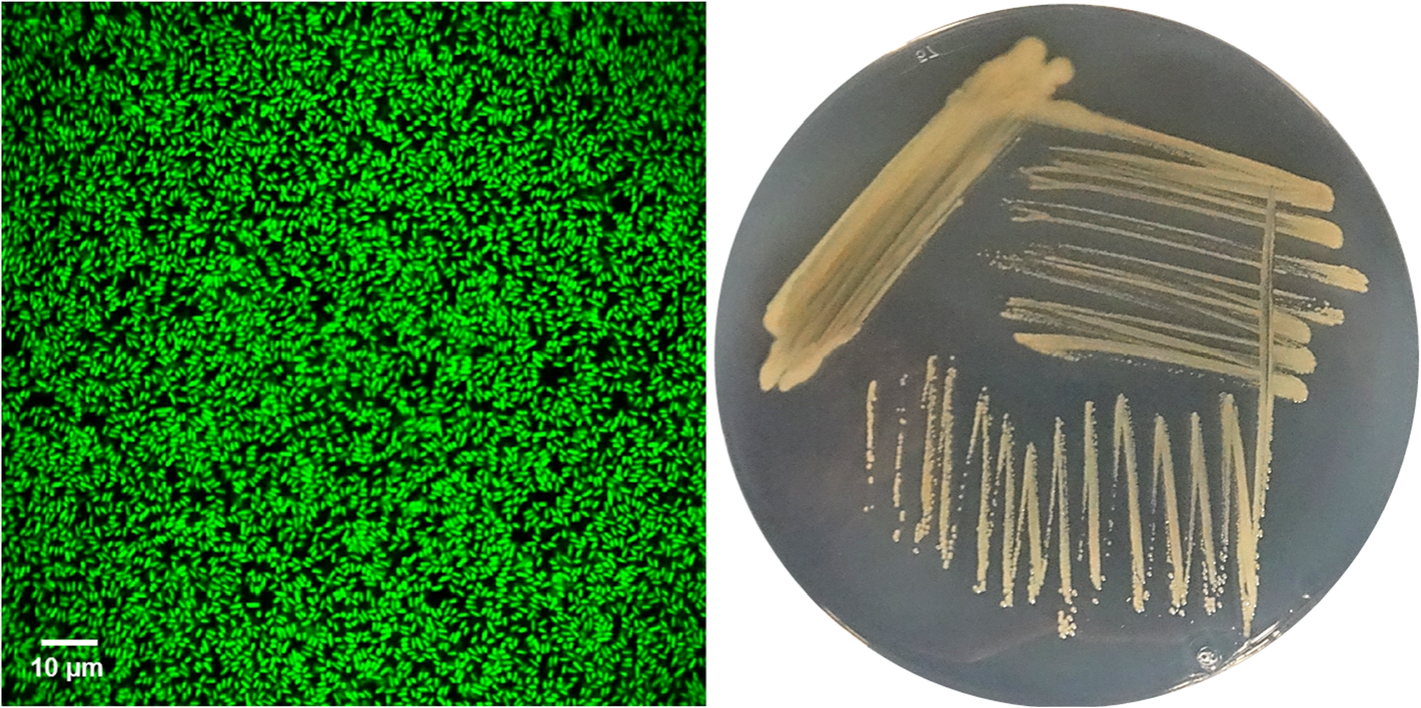
Photomicrographs of source organism. Image of *P. corrugata* RM1-1-4 cells using confocal laser scanning microscopy (CLSM, left) and the appearance of colony morphology after 48 h growing on NB agar medium at 25 °C (right). Image was obtained using acridin orange (0.4 mg mL^−1^ water) stained RM1-1-4 cells with 40× magnification. Cells under Leica TCS SP CLSM (Leica Microsystems, Wetzlar, Germany) captured and analysed using Leica Application Suite Advanced Fluorescence (LAS AF) software Version 3.5

10.1601/nm.2592 RM1-1-4 is a motile, Gram-negative, non-sporulating rod in the order 10.1601/nm.2550 of the class 10.1601/nm.2068. The rod-shaped cells are approximately 0.5 μm in width and 1.5–2.0 μm in length (Fig. 1 left). The strain is moderately fast-growing, forming 1 mm colonies within 1–2 days at 25 °C. Colonies formed on nutrient broth II (NBII) agar plates [1] are yellow opaque shining, domed and moderately mucoid with smooth margins (Fig. 1 right). No fluorescence of the cells was visualized under UV light (312 nm) when grown on King’s B agar. RM1-1-4 was isolated from the roots of healthy oilseed rape plants cv. Traviata KWS (KWS SAAT SE, Einbeck, Germany), whose seeds were treated with a microbial suspension of *Sphagnum magellanicum* [1].

Even though the optimal growth temperature is 30 °C, RM1-1-4 can also slowly replicate at 5 °C in liquid Luria Bertani (LB). Growth was observed at 37 °C and slightly at 40 °C in this culturing medium and on solidified medium after 24 h. The strain grows in complex media (LB, NBII), but not in Standard Succinate Medium (pH 7.0). Optimum pH for growth in LB is pH 6.0. It does not cause any deleterious effect on its original host (oilseed rape) or maize, sorghum and sugar beet [1]. Strain RM1-1-4 has natural resistance to gentamycin (10 μg mL^−1^), trimethoprim (50 μg mL^−1^) and is able to develop spontaneous rifampicin-resistance.

**Fig. 2:**
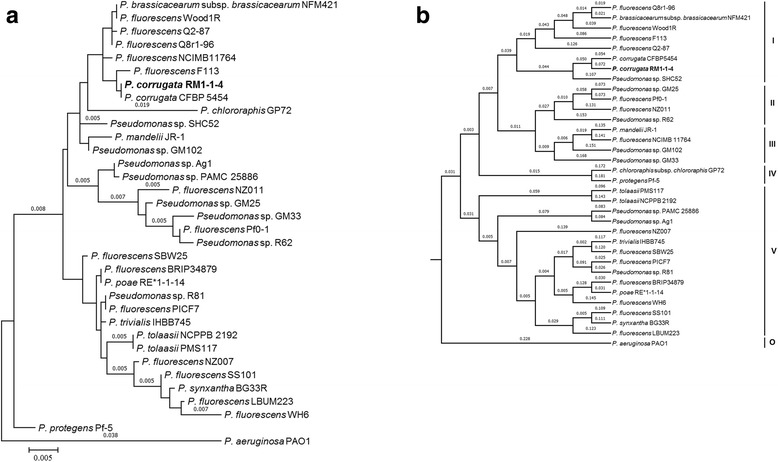
Phylogenetic tree showing the position of *P. corrugata* RM1-1-14 in relationships among other strains of *Pseudomonas* spp. including *P. aeruginosa* PAO1 as outgroup. **a** The tree is based on 16S rRNA gene alignments and was conducted in MEGA6 [3]. Initial tree for the heuristic search were obtained automatically by applying Neighbor-Join and BioNJ algorithms to a matrix of pairwise distances estimated using the Maximum Composite Likelihood approach, and then selecting the topology with superior log likelihood value. **b** The dendrogram based on protein sequences for representative strains belonging to the existing five subgroups (I-V) of the *P. fluorescens* complex including *P. aeruginosa* PAO1 as outgroup (O) and was created by using Composition Vector Approach [5]

Minimum Information about the Genome Sequence (MIGS) of 10.1601/nm.2592 RM1-1-4 is summarized in Table 1. The phylogenetic relationship of 10.1601/nm.2592 RM1-1-4 to other species within the genus 10.1601/nm.2552 is visualized in a 16S rRNA based tree [3] and a tree based on the oligopeptide content of the complete protein sequence by using a Composition Vector Tree (CVTree) approach [4, 5] (Fig. 2a, b).

**Table 1:** Classification and general features of *P. corrugata* RM1-1-4 according to the MIGS recommendation [24]

MIGS ID	Property	Term	Evidence code^a^
	Classification	Domain *Bacteria*	TAS [25]
		Phylum *Proteobacteria*	TAS [26]
		Class *Gammaproteobacteria*	TAS [27]
		Order *Pseudomonadales*	TAS [28, 29]
		Family *Pseudomonadaceae*	TAS [26, 30]
		Genus *Pseudomonas*	TAS [31–34]
		Species *Pseudomonas corrugata*	TAS [34]
		Strain: RM1-1-4	TAS [1]
	Gram stain	Negative	IDA, TAS [34]
	Cell shape	Rod-shaped	IDA, TAS [34]
	Motility	Motile	TAS [34]
	Sporulation	None	NAS
	Temperature range	5 °C–40 °C	IDA
	Optimum temperature	30 °C	IDA
	pH range; Optimum	5–9; 6	IDA
	Carbon source	Heterotrophic	TAS [34]
MIGS-6	Habitat	Soil, Rhizosphere	TAS [1]
MIGS-6.3	Salinity	1–9% NaCl (*w*/*v*)	IDA, TAS [1]
MIGS-22	Oxygen requirement	Aerobic	TAS [34]
MIGS-15	Biotic relationship	Rhizospheric	TAS [1]
MIGS-14	Pathogenicity	Non-pathogen	TAS [1]
	Host	*Brassica napus* L.	TAS [1]
	Host taxa ID	3708	
	Biosafety level	1	NAS
MIGS-4	Geographic location	Graz, Austria	TAS [1]
MIGS-5	Sample collection time	2010	TAS [1]
MIGS-4.1	Latitude	47.065545	NAS
MIGS-4.2	Longitude	15.453093	NAS
MIGS-4.4	Altitude	1340 m	NAS

^a^Evidence codes - IDA: Inferred from Direct Assay; TAS: Traceable Author Statement (i.e., a direct report exists in the literature); NAS: Non-traceable Author Statement (i.e., not directly observed for the living, isolated sample, but based on a generally accepted property for the species, or anecdotal evidence). These evidence codes are from the Gene Ontology project [35]

## Genome sequencing information

### Genome project history

The genome of 10.1601/nm.2592 strain RM1-1-4 was selected for sequencing based on its ability to exert stress protecting abilities against abiotic and biotic stresses and to promote plant growth. The strain was isolated from the rhizosphere of an oilseed rape plant that was subjected to a bait plant strategy: oilseed rape seeds were treated with the microbial community of *Sphagnum magellanicum* [1], where RM1-1-4 was originally identified as 10.1601/nm.2606. After Average Nucleotide Identity (ANI) [6] comparison of the genome sequence against the genomes of the type strains and proxytype strains that are already in GenBank, RM1-1-4 showed 99.585% identity to the type genome of 10.1601/nm.2592 with 97% coverage of the genome. To clarify the taxonomic affiliation we performed a systematic method of inferring evolutionary relatedness of microbial organisms from the 16S rRNA gene region (Fig. 2a) and the oligopeptide content of their complete protein sequences by using CVTree showing its phylogenetic positioning (Fig. 2b) [3–5]. The genome project is deposited in the NCBI BioProject PRJNA309490 database with the Biosample SAMN04453325. This whole genome shotgun project has been deposited in the NCBI database under the accession no. CP014262 (Table 2).

**Table 2:** Project information

MIGS ID	Property	Term
MIGS 31	Finishing quality	Finished
MIGS-28	Libraries used	PacBio RS libraries with inserts of 8 to 20 kb
MIGS 29	Sequencing platforms	PacBio RS II sequencer
MIGS 31.2	Fold coverage	118.0
MIGS 30	Assemblers	Hierarchical Genome Assembly Process (HGAP) algorithm implemented in the PacBio SMRT Analysis software
MIGS 32	Gene calling method	Glimmer gene prediction, NCBI Prokaryotic Genome Annotation Pipeline
	Locus Tag	AXG94
	Genbank ID	CP014262
	GenBank Date of Release	July 31, 2016
	GOLD ID	Gs0118516, Gp0137000, Ga0115603
	BIOPROJECT	PRJNA309490
MIGS 13	Source Material Identifier	RM1-1-4
	Project relevance	Plant-bacteria interaction, agricultural, environmental

### Growth conditions and genomic DNA preparation

10.1601/nm.2592 strain RM1-1-4 was grown in 50 mL of NBII (Sifin, Berlin, Germany) medium and incubated for 20 h at 30 °C. 0.5 mL were centrifuged at 2500 x g for 5 min at 4 °C and genomic DNA was extracted using the MasterPure DNA purification kit (Epicentre, Madison, WI, USA). DNA quality and quantity were checked by agarose gel electrophoresis and spectrophotometry using a UV-Vis spectrophotometer (NanoDrop 2000c, Thermo Fisher Scientific, Waltham, MA USA). In total, 91 μg genomic DNA (3.1 μg μL^−1^) was sent on dry ice to the sequencing service. PacBio RS libraries with inserts of 8 to 20 kb were constructed and sequenced at GATC Biotech (Konstanz, Germany).

### Genome sequencing and assembly

PacBio RS libraries with inserts of 8 to 20 kb were constructed and sequenced at GATC Biotech (Konstanz, Germany) using single molecule, real-time (SMRT) sequencing. Assembly was completed with the Hierarchical Genome Assembly Process (HGAP) algorithm implemented in the PacBio SMRT Analysis software (Pacific Biosciences, Menlo Park, CA, USA). The assembly of RM1-1-4 genome based on 161,326 quality reads with a mean length of 5315 bp resulting in a single circular chromosome of 6,124,363 bp, with 118.0-fold overall coverage and a GC content of 60.7%.

### Genome annotation

Automatic annotation was conducted on the RAST Web server (version 36) using RAST gene calling based on FIGfam version Release70 [7, 8], and additional annotation for using the automated assignment of COG-functions to protein-coding genes was completed on the BASys web server using Glimmer gene prediction [9, 10]. Pseudogenes were predicted using the NCBI Prokaryotic Genome Annotation Pipeline. Signal peptides and transmembrane helices were predicted using SignalP [11, 12].

## Genome properties

**Fig. 3:**
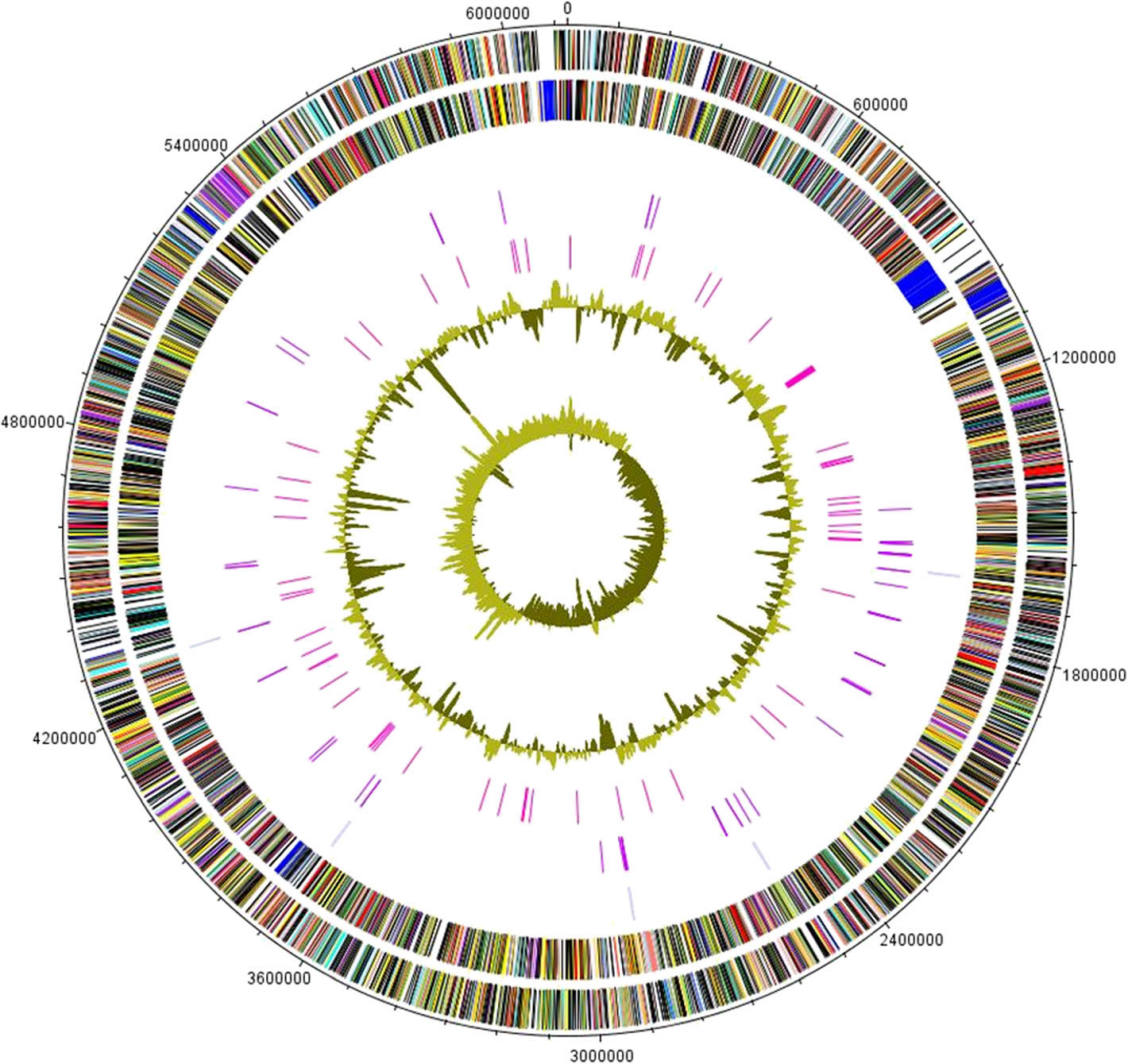
Graphical map of the chromosome. The outer scale is marked every 10 kb. Circles range from 1 (outer circle) to 7 (inner circle). Circle 1 and 2, ORFs encoded by leading and lagging strand respectively, with color code for functions: salmon, translation, ribosomal structure and biogenesis; aquamarine, RNA processing and modification; light blue, transcription; cyan, DNA replication, recombination and repair; tan, chromatin structure and dynamics; turquoise, cell division; dark orange, defense mechanisms; deep pink, post-translational modification, protein turnover and chaperones; dark olive green, cell envelope biogenesis; purple, cell motility and secretion; lavender, intracellular trafficking, secretion, and vesicular transport; forest green, inorganic ion transport and metabolism; pink, signal transduction; red, energy production; sienna, carbohydrate transport and metabolism; yellow, amino acid transport; orange, nucleotide transport and metabolism; gold, co-enzyme transport and metabolism; cornflower blue, lipid metabolism; blue, secondary metabolites, transport and catabolism; gray, general function prediction only; yellow green, unknown function; black, function unclassified or unknown. Circle 3 and 4, distributions of tRNA genes and rrn operons respectively. Circle 5, distribution of pseudogenes. Circle 6 and 7, G + C content and GC skew (G-C/G + C) respectively

The genome of RM1-1-4 is composed of one circular chromosome consisting of 6,124,363 bp with an average GC content of 60.7% (Table 3 and Fig. 3), which is comparable to that of other 10.1601/nm.2592 strains. Among the 5335 predicted genes, 5189 were identified as protein coding genes of which 4110 (79.2%) were assigned as putative function, while the other 1079 (20.8%) were designated as hypothetical proteins. The classification of CDSs into functional categories according to the COG (Clusters of Orthologous Groups) [13, 14] database is summarized in Table 4 on BASys gene prediction. Beside the predicted genes, the genome annotation revealed 65 tRNA, five rRNA loci (5S, 16S, 23S) with one additional 5S rRNA, four ncRNAs and 284 predicted SEED subsystem features.

**Table 3:** Genome statistics

Attribute	Value	% of Total
Genome size (bp)	6,124,363	100
DNA coding (bp)	5,492,379	89.7
DNA G + C (bp)	3,715,247	60.7
DNA scaffolds	1	–
Total genes	5335	100
Protein coding genes	5189	97.3
RNA genes	85	1.6
Pseudo genes	61	1.1
Genes in internal clusters	NA	–
Genes with function prediction	4256	82.0
Genes assigned to COGs	4013	77.3
Genes with Pfam domains	3296	63.5
Genes with signal peptides	434	8.4
Genes with transmembrane helices	1365	26.3
CRISPR repeats	NA	–

**Table 4:** Number of genes associated with general COG functional categories

Code	Value	%age	Description
J	161	2.41	Translation, ribosomal structure and biogenesis
A	3	0.04	RNA processing and modification
K	375	5.61	Transcription
L	162	2.43	Replication, recombination and repair
B	2	0.03	Chromatin structure and dynamics
D	32	0.48	Cell cycle control, Cell division, chromosome partitioning
V	61	0.91	Defense mechanisms
T	235	3.52	Signal transduction mechanisms
M	236	3.53	Cell wall/membrane biogenesis
N	126	1.89	Cell motility
U	42	0.63	Intracellular trafficking and secretion
O	168	2.52	Posttranslational modification, protein turnover, chaperones
C	270	4.04	Energy production and conversion
G	247	3.70	Carbohydrate transport and metabolism
E	445	6.66	Amino acid transport and metabolism
F	79	1.18	Nucleotide transport and metabolism
H	152	2.28	Coenzyme transport and metabolism
I	182	2.72	Lipid transport and metabolism
P	197	2.92	Inorganic ion transport and metabolism
Q	97	1.45	Secondary metabolites biosynthesis, transport and catabolism
R	412	6.17	General function prediction only
S	329	4.93	Function unknown
–	2615	40.14	Not in COGs

The total is based on the total number of protein coding genes in the genome based on BASys gene prediction

## Insights from the genome sequence

The genome encodes genes that can be linked to detoxification mechanisms of oxygen radicals, toxins and heavy metals by efflux pumps as well as to stress response by heat and cold shock proteins and the universal stress protein A (UspA). UspA with orthologues (Locus Tags AXG94_02180, AXG94_04130, AXG94_24005, AXG94_24695) could play a significant role in protecting RM1-1-4 cells from H_2_O_2_ and low pH as found in organisms inhabiting extreme environments [15] and analysed in detail for the clinical strain 10.1601/nm.2767 10.1601/strainfinder?urlappend=%3Fid%3DATCC+17978 [16]. A water stress/hypersensitive response protein (AXG94_21760) is present, which is supposed to be transferred to symbiotic or pathogenic bacteria by horizontal gene transfer from plants and can be seen as the acquisition of a function putatively related to the cell defense [17]. The genome of RM1-1-4 contains several genes, which are important contributors to biological control. They are related to the biosynthesis of secondary metabolites or antimicrobial products that are similar to those found in the genomes of other Pseudomonads: productions of exoproteases and lipoproteins [18]. We further identified genes most probably involved in the direct promotion of plant growth, such as biosynthesis or carrier gene clusters for aminocyclopropane-1-carboxylate deaminase suggested to be a key in the modulation of ethylene levels in plants by bacteria [19], auxin, biofilm dispersion, rhizobactin siderophores and spermidine.

Genes predicting the synthesis of volatile components are present in the RM1-1-4 genome as well. Volatile components have been shown to act as antibiotics and to induce plant growth [20, 21]. An example is hydrogen cyanide (HCN), an inorganic compound with antagonistic effects against soil microbes [22]. RM1-1-4 encodes a hydrogen cyanide synthase HcnA (AXG94_04380) and orthologues of genes required for the biosynthesis of other volatile components such as 2,3-butanediol (AXG94_01200) and its precursor acetoin (AXG94_01195) were annotated too. Beside the presence of specific genes and the noticeable ability of RM1-1-4 to expose stress protection, the function of particular genes needs to be clarified in further detailed studies.

The genome-wide phylogenetic analysis on 10.1601/nm.2552 species [3–5] with the RM1-1-4 genome showed that strain RM1-1-4 clusters within the 10.1601/nm.2606 group (Fig. 2a, b) and most closely to 10.1601/nm.2592 10.1601/strainfinder?urlappend=%3Fid%3DCFBP+5454 (DDBJ/EMBL/GenBank accession ATKI01000000). The two 10.1601/nm.2592 strains belong to the few non fluorescent 10.1601/nm.2552 species. 10.1601/strainfinder?urlappend=%3Fid%3DCFBP+5454 was originally described as the causal agent of the tomato disease called ‘pith necrosis’ and is yet considered as a biological resource in the fields of biocontrol of plant diseases and production of industrially promising microbial biopolymers like antimicrobial cyclic lipopeptides [23].

## Conclusions

This report described the complete genome sequence of 10.1601/nm.2592 strain RM1-1-4. It is a “10.1601/nm.2550” within the non-fluorescent 10.1601/nm.2592 clade that was originally isolated from the roots of moss microbiome-primed oilseed rape seeds cv. Traviata KWS grown in a greenhouse in Graz, Austria. This strain was selected for sequencing based on its ability to protect plants from abiotic and biotic stresses and to promote plant growth. We could highlight genes encoding abiotic and biotic stress protecting factors and other well-known bacterial traits for establishment of beneficial plant-microbe interactions. The genome encodes for a collection of genes predicting biofilm dispersion, detoxifying compounds, volatile components and enzymes such as a protease and a deaminase. Such properties likely have origins in a repertoire of genes including efflux pumps, putative T2SS, T4SS and T6SS, and several genes presumably implicated in auxin, rhizobactin siderophore and spermidine production. Further functional studies and comparative genomics with related isolates will provide insights into naturally acquired plant stress protection and promotion of plant health.

## Abbreviations

CDS: Coding DNA Sequence
CLSM: Confocal Laser Scanning Microscopy
COG: Clusters of Orthologous Groups
CVTree: Composition Vector Tree
HCN: Hydrogen Cyanide
HGAP: Hierarchical Genome Assembly Process
LB: Luria Bertani
NBII: Nutrient Broth II
RAST: Rapid Annotations using Subsystems Technology
SMRT: Single Molecule, Real-Time
T2SS: Type 2 Secretion System
